# Somatostatin Analogs Therapy in Gastroenteropancreatic Neuroendocrine Tumors: Current Aspects and New Perspectives

**DOI:** 10.3389/fendo.2014.00007

**Published:** 2014-02-07

**Authors:** Roberto Baldelli, A. Barnabei, L. Rizza, A. M. Isidori, F. Rota, P. Di Giacinto, A. Paoloni, F. Torino, S. M. Corsello, A. Lenzi, M. Appetecchia

**Affiliations:** ^1^Endocrinology Unit, Regina Elena National Cancer Institute, Rome, Italy; ^2^Section of Endocrinology, Department of Experimental Medicine, “Sapienza” University of Rome, Rome, Italy; ^3^Section of Reproductive Endocrinology, Department of Systems Medicine, Tor Vergata University of Rome, Fatebenefratelli Hospital, Rome, Italy; ^4^Department of Systems Medicine, Tor Vergata University of Rome, Rome, Italy; ^5^Department of Endocrinology, Catholic University of the Sacred Heart, Rome, Italy

**Keywords:** neuroendocrine tumors, somatostatin analogs, lanreotide, octreotide, carcinoid

## Abstract

Gastroenteropancreatic neuroendocrine tumors (GEP-NETs) are rare tumors that present many clinical features secreting peptides and neuroamines that cause distinct clinical syndromes such as carcinoid syndrome. However most of them are clinically silent until late presentation with mass effects. Surgical resection is the first line treatment for a patient with a GEP-NET while in metastatic disease multiple therapeutic approaches are possible. GEP-NETs are able to express somatostatin receptors (SSTRs) bounded by somatostatin (SST) or its synthetic analogs, although the subtypes and number of SSTRs expressed are very variable. In particular, SST analogs are used frequently to control hormone-related symptoms while their anti-neoplastic activity seems to result prevalently in tumor stabilization. Patients who fail to respond or cease to respond to standard SST analogs treatment seem to have a response to higher doses of these drugs. For this reason, the use of higher doses of SST analogs will probably improve the clinical management of these patients.

## Introduction

**Figure 1:**
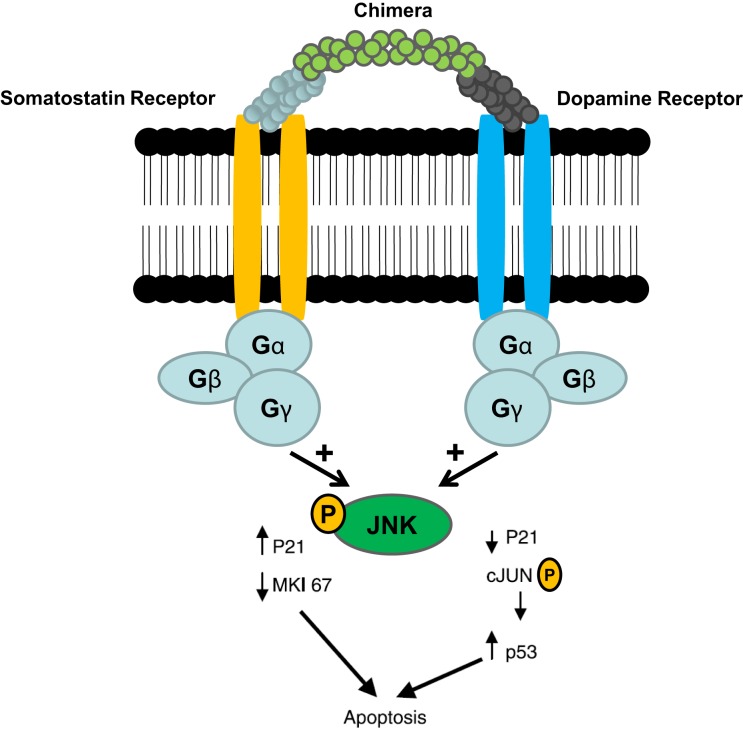
**Somatostatin/dopamine chimera-induced dimerization of somatostatin and dopamine receptors** [Adapted from Ref. (11)].

Gastroenteropancreatic neuroendocrine tumors (GEP-NETs) are characterized by an yearly incidence of 1.2–3.0 cases/100,000 inhabitants (1–5). The majority of the GEP-NETs are sporadic but they can be also part of familiar syndromes such as MEN 1 syndrome, von Hippel–Lindau disease, and neurofibromatosis type 1 while the clinical characteristics depend on the site of the primary tumor and its ability to secrete neuroamines and peptides. Among functioning tumors, major clinical entities are represented by carcinoid syndrome, hypoglycemic syndrome, Zollinger–Ellison syndrome, WDHA (Water Diarrhea–Hypokalemia–Achlorydria) syndrome, and glucagonoma syndrome. However most of the GEP-NETs are not able to produce biologically active hormones (non-functioning tumors) and therefore the diagnosis is often made too late only for the presence of symptoms due to the mass effect and/or metastases, mainly hepatic (1). In patients with localized well-differentiated neuroendocrine carcinomas, 5-year-survival is 60–100% while with regional disease or distant metastases 5-year-survival is 40 and 29%, respectively (6). Around 80% of GEP-NETs express somatostatin receptors (SSTRs); they are five different G-protein coupled receptor subtypes (SSTRs 1–5) that are differently expressed in the various types of tumor (Tables 1 and 2). It is important to underline that SSTRs are present not only in neoplastic tissues. For example Beneyto and co-workers used *in situ* hybridization to quantify the mRNA expression levels of SST receptors subtype 1 (SSTR1) and subtype 2 (SSTR2) in dorsolateral prefrontal cortex area 9 from 23 matched pairs of subjects with schizophrenia and normal comparison subjects. SSTR1 mRNA levels did not differ between subject groups while mean cortical SSTR2 mRNA levels were significantly 19% lower in the subjects with schizophrenia (7). Moreover in a very interesting and complete work, Pasquali and co-workers, reported that the radiolabeled somatostatin (SST) analog octreotide accumulates within the orbits of active Graves’ ophthalmopathy (GO), and octreotide and lanreotide have been proposed to treat this disorder. In particular, the authors described the expression of SST1–5 genes in lymphocytes recovered from retroorbital tissues obtained from patients with GO undergoing orbital decompression. All SSTs transcripts were found in lymphocytes both from GO retroorbital tissues and blood samples (8). In addition, recent studies have shown that SSTRs are preferably expressed in well-differentiated neoplasia and some advanced forms loose particular receptor subtypes while keeping others (9, 10); SSTRs subtypes can form homo/heterodimers at the membrane level, developing new receptors with different functional features (11), and that this receptor dimerization may be induced by addition of either dopamine or SST (Figure 1). In a study examining 81 functioning and non-functioning GEP-NETs the large part of the tumors expressed SSTRs 1, 2, 3, and 5, while SSTR 4 was detected only in a small minority (12). SSTRs have been extensively mapped in different pancreatic tumors by means of autoradiography, reverse-transcription polymerase chain reaction, *in situ* hybridization, and immunohistochemistry; SSTRs 1, 2, 3, and 5 are usually expressed in pancreatic NETS in particular insulinomas had heterogeneous SSTRs expression while 100% of somatostatinomas expressed SSTR 5 and 100% of gastrinomas and glucagonomas expressed SSTR 2 (13). SST is a natural peptide hormone secreted in various parts of the human body, including the digestive tract, able to inhibit the release of numerous endocrine hormones, including insulin, glucagon, and gastrin. The biological effects of SST are mediated through its specific receptors (SSTRs 1–5) all bind natural peptides (SST-14, SST-28, and cortistatin) with similar high affinity. However, endogenous SST short half-life in circulation (1–3 min), makes it difficult to use it continuously and has resulted in the development of synthetic analogs from the early 1980s when a number of short synthetic analogs of SST including SMS201-995 (octreotide), RC-160 (vapreotide), BIM 23014 (lanreotide), and MK 678 (seglitide) were developed. These cyclic octapeptides are more resistant to peptidases and their half-lives and hence their biological activities are substantially longer than native SST (1.5–2 h vs. 1–2 min) (14). Moreover the development of a depot formulation of octreotide [octreotide long-acting repeatable (LAR)], administered up to 30 mg once every 4 weeks has to a large extent eliminated the need for daily injections. Lanreotide SR (slow release) 30, 60, and 90 mg formulations administered every 10–14 days, has a similar efficacy to octreotide in the treatment of carcinoid tumors (15). A new slow release depot preparation of lanreotide, lanreotide autogel, is administered subcutaneously up to 120 mg once a month (16). Native SST and its synthetic analogs show different affinity for the five specific SSTRs (11, 12, 17). Native SST binds all the five receptor subtypes (SSTRs 1–5). The effects of the SST analogs are mediated by the interaction with SSTR 2 and 5 receptors while the new SST analog, pasireotide (SOM 230), shows higher binding capacity toward SSTRs 1, 2, 3, and 5 with no agonist activity at the type 4 receptor (17) (Table 3). Moreover, *in vitro* studies demonstrated that SOM 230 was more effective than octreotide to control cell proliferation and apoptosis (18). The different receptor subtypes binding affinities seems to result in different biological and clinical activities (12).

**Table 1:** **Somatostatin receptors^a^ in neuroendocrine gastroenteropancreatic tumors (%)**.

	SSTR1	SSTR2	SSTR3	SSTR4	SSTR5
All	68	86	46	93	57
Insulinoma	33	100^b^	33	100	67
Gastrinoma	33	50	17	83	50
Glucagonoma	67	100	67	67	67
VIPoma	100	100	100	100	100
N-F	80	100	40	100	60

*IP, vasoactive intestinal polypeptide; N-F, non-functioning*.

*^a^Using receptor subtype antibodies*.

*^b^Malignant insulinoma*.

*Modified from Oberg et al. (15)*.

**Table 2:** **Somatostatin receptor subtypes mRNA in neuroendocrine tumors**.

Tumor	SST1 (%)	SST2 (%)	SST3 (%)	SST4 (%)	SST5 (%)
Gastrinoma	79^a^	93	36	61	93
Insulinoma	76	81	38	58	57
Non-functioning pancreatic tumor	58	88	42	48	50
Carcinoid tumor of the gut	76	80	43	68	77

*SST, somatostatin receptor*.

*^a^Indicates the percentage of positive tumors for each SSTRs mRNA expression may overestimate the number of receptors present, depending on the technique used (PR-polymerase chain reaction, Northern blot, *in situ* hybridization)*.

*Modified from Plockinger (19)*.

**Table 3:** **Somatostatin receptor subtype-binding affinity of somatostatin analogs**.

Compound	SSTR1	SSTR2	SSTR3	SSTR4	SSTR5
**RECEPTOR SUBTYPE AFFINITY (IC50, nM)**					
Somatostatin-14	2.26	0.23	1.43	1.77	0.88
Somatostatin-28	1.85	0.31	1.3	ND	0.4
Octreotide	1140	0.56	34	7030	7
Lanreotide	2330	0.75	107	2100	5.2
Pasireotide	9.3	1	1.5	>100	0.16

*ND, not determined*.

*Modified from Ref. (20)*.

## The Symptomatic and Biochemical Effects of SST Analogs

Gastroenteropancreatic neuroendocrine tumors first line therapy, where possible, is always an aggressive surgical approach, aimed to obtain a curative tumor ablation, even in the presence of metastatic disease. However, in patients with functioning or metastatic tumors, the treatment goal is to improve their quality of life trying to alleviate the tumor associated symptoms and increasing survival (2, 14, 15). Recently, the diagnostic and therapeutic approach of GEP-NETs has considerably improved, mainly due to better imaging techniques (CT, MRI, PET) and SST analog-based imaging methods, as well as receptor subtype characterization and the introduction of long-acting SST analogs (21–27). Usually, the treatment with long-acting preparations of SST analogs consists in an intramuscular injection (i.m.) every 2 or 4 weeks (octreotide-LAR, 10–30 mg; lanreotide autogel 60–120 mg) and the efficacy in the control of symptoms is well-documented (2, 14, 15), even if patients with islet cell tumor often show a transient (median time 2.5 months) and non-significant response (21–27). The drugs are safe and well-tolerated in both long- and short-term treatments (28–32). However, after 9–12 months, drug resistance should appear and patients may show the symptoms of recrudescence. In such cases the approach proposed was to continue the treatment by increasing the analog dosage or by shortening the administration range (33). A randomized double-blind trial compared long-acting octreotide-LAR at 10, 20, and 30 mg every 4 weeks with open-label short-acting octreotide every 8 h for the treatment of carcinoid syndrome. It showed that the efficacy of short-acting octreotide and of the long-acting octreotide-LAR was the same once circulating octreotide steady-state concentrations were achieved (34). O’Toole et al. in a multicentre study on 33 patients with the carcinoid syndrome comparing the treatment with lanreotide (30 mg i.m. every 10 days) vs. octreotide (200 μg s.c. twice or thrice daily) founded no significant differences in controlling symptoms (53.8 and 45.4%, respectively). Lanreotide and octreotide may also significantly lower the levels of the catabolite of serotonin (urinary 5-hydroxyindoleacetic acid/5-HIAA) (35). Ruszniewski et al. evaluated the efficacy and safety of the 28-day-aqueous prolonged release formulation of lanreotide in 75 patients in a 6-month-dose titration study where 30% of patients showed a biochemical response and 75 and 80% of patients reported resolution of diarrhea and flushing, respectively. The median decrease in levels of urinary 5-HIAA and serum chromogranin A was 24 and 38%, respectively (36). An interim analysis of a phase II trial of SOM230 in 21 patients with metastatic carcinoid tumors whose symptoms (diarrhea and flushing) were refractory/resistant to octreotide-LAR showed symptom relief in 33% (37). Approximately 10–15% of patients with midgut carcinoids suffer from watery diarrhea, flushing, right-sided heart failure, and bronchial constriction (carcinoid syndrome), due to the tumor hypersecretion of a variety of endocrine substances, the most frequent of which are serotonin (5-hydroxytryptamine) and the tachykinins (38, 39), and therefore SST analogs are important palliative tools for these patients. In insulinoma it has been noted that octreotide treatment may induce hypoglycemia worse in those patients lacking SSTRs 2 and 5, and as glucagon secretion is also inhibited, patients have to be observed closely at the beginning of therapy to prevent severe hypoglycemia due to the reduced glucagon-dependent counter-regulation (19). Hence, this treatment has to be started in a hospital setting, and should be reserved only for the minority of insulinoma patients with positive imaging on SRS. Vezzosi et al. recently assessed that octreotide was effective in the control of hypoglycemia in more than 50% of the insulinoma patients (40). The treatment was effective in all SSTR 2 positive patients and in a few SSTR 2 negative ones, while no relation between treatment effectiveness and the expression of SSTR 5 was observed (40). These results are in concordance with other case reports and smaller series of insulinoma patients reported in the literature (41–45). In glucagonoma patients SST analog treatment is indicated to reduce the symptoms related to the characteristic skin rash (necrolytic migratory erythema) or diarrhea (46–50). Somatostatinomas symptoms are due to SST hypersecretion (hyperglycemia, cholelithiasis, diarrhea and steatorrhea, and hypochlorhydria) or to the mass effect (51). Although it seems a paradox to treat patients with symptoms related to elevated SST levels with a somatostatinoma, in 1998 Angeletti et al. showed that octreotide treatment was effective in reducing SST plasma levels and improving the related symptoms in three patients with metastatic somatostatinomas (52). Furthermore, nine cases of VIPoma have been described in which octreotide was very successful as adjuvant therapy for symptoms control and for reducing the serum elevated VIP levels by improving the diarrhea and the electrolyte imbalance (53–55).

## The Anti Tumor Effects of SST Analogs

The anti-neoplastic activity of SST analogs has been demonstrated in several experimental models *in vivo* and *in vitro* (56–61) but it is still little known regarding the anti-proliferative role of SSA in GEP-NETs, although increasing data suggest that such analogs can be tumoristatic, at least in some circumstances (62). The anti-neoplastic action of SST analogs depends on the kind of tumor and the receptor subtypes to which they are bound to and occurs through direct and indirect mechanisms. While direct activities are mediated by specific membrane receptors and include antimytotic and apoptotic effects, indirect effects do not depend on the receptor binding but depend on the growth factor inhibition, antiangiogenic, and immuno-modulating activities. SST analogs are able to inhibit the growth of Swarm chondrosarcoma, used as experimental model of SSTR free tumor (63). The mitosis inhibition is mediated by SSTRs 2 and 5 and results in the cell cycle arrest (56). The loss of the SSTR 2 expression in some human adenocarcinomas seems to be responsible for loosing the regulation of cell proliferation (10–19, 21–64). The loss of SSTR 2 may consequently promote tumor growth and make it clear the therapeutic inefficacy of SST analogs in such kind of neoplasia. Apoptosis seems to be induced by two different processes: interaction with the SSTR 3 (57) and inhibition of the Insulin-like Growth Factor I (IGF I), known as a potent antiapoptotic hormone (65). The pro-apoptotic activity of SST analogs seems to have clinical relevance, as shown by the interesting findings published by Eriksson et al. that reported an increase in apoptosis in bioptic samples of tissues by patients with GEP-NETs after the treatment with SST analogs at high doses. It followed that apoptosis is related to the biochemical response and the disease stabilization (70% of cases) (66, 67). However, Faiss et al. observed an overall response rate (ORR) of 6.7%, comparable to that recorded at conventional doses (68), in 24 patients with GEP-NETs treated with high doses of lanreotide (15 mg/day). The indirect anti-proliferative efficacy of SST analogs is shown by an antiangiogenic mechanism. Tumor angiogenesis is essential in the development and metastatic spread of tumors, so the growth can be actually controlled by reducing the vascularization of the neoplastic tissue. In experimental models, octreotide shows a strong antiangiogenic effect, which is probably mediated by the inhibition of the Vascular Endothelial Growth Factor (VEGF) (69–71). The treatment with octreotide would result in a significant reduction in VEGF levels compared to the baseline, since it is related to the survival of the patients (71). It was observed that endothelial cells do not express the SSTR 2 that is present on the contrary, when they proliferate in order to form blood vessels. This could represent further opportunity to treat patients with octreotide that is able to recognize and inhibit new vessel formation both alone and with other drugs, thanks to its high affinity with such receptor (Table 3). Immunomodulation is another indirect mechanism of action of SST analogs. Preliminary evidence suggests that they stimulate the production of immune system components with antitumor effect, such as *natural killer* cells (72, 73), even if up to now it is not clear whether this can be clinically significant thus helping the antitumor efficacy of SST analogs. Few data exist on the functions mediated by the SSTR 4. However, no unanimity exists about the SST analog ability to control (i.e., to slow) the tumor progression. *In vitro* studies reported that the response of different cell lines to the octreotide exposition produces a biphasic dose–response curve (74, 75). Consequently, overdose or underdose of SST analogs may result in a suboptimal anti-neoplastic activity. Nevertheless, the negative results of some clinical studies in terms of tumor response could be due to the administration of too low doses to achieve receptor optimal saturation. After all, in other studies that used octreotide doses higher than 8 mg/day and lanreotide doses higher than 10 mg/day (76), no improvement of the SST analog antitumor effect was observed. No study on the tumor response monitored plasma levels of an SST analog up to now, in order to assess that optimal drug therapeutic levels are reached but not exceeded (77). Tumor shrinkage was demonstrated in <10% of the patients. However, a stabilization of tumor growth occurs in up to 50% of the patients with neuroendocrine tumors of various locations. Stable disease was observed in 37–45% of the patients with documented tumor progression before SSA therapy (Table 4). The median duration of stabilization was 26.5 months (31, 78–81). In a study on a selected group of patients with progressive disease, in the 47% of cases was demonstrated a stable disease when treated with a high dose of lanreotide (3–5 g/day) (82). This result has been confirmed in patients with advanced midgut carcinoids, who had a stabilization of the disease for 6–24 months in the 75% of cases (83). One patient with a pancreatic primary tumor and distant extrahepatic metastases, showed a poor response to treatment in multivariate analysis. Age, size of the primary tumor, and Ki67 did not influence the response rate to SSA therapy (81). A stabilization of the disease was maintain throughout long-term follow-up in patients who achieve it after 6 months of treatment; these patients live longer than those unresponsive to therapy (81, 84). Very recently, Rinke et al performed for the first time a placebo-controlled, double-blind, and phase III B study in 85 patients with well-differentiated metastatic midgut NETs using octreotide-LAR 30 mg intramuscularly in monthly intervals (PROMID study). Median time to tumor progression in the octreotide-LAR and placebo groups was 14.3 and 6 months, respectively. After 6 months of treatment, stable disease was observed in 66.7% of patients in the octreotide-LAR group and 37.2% of patients in the placebo group. Functionally active and inactive tumors responded similarly. The most favorable effect was observed in patients with low hepatic tumor load and resected primary tumor. Octreotide-LAR significantly lengthened the time to tumor progression compared with placebo in patients with functionally active and inactive metastatic midgut NETs (85). Midgut carcinoids express SSTRs in 80–100% of cases and SSTR 2 is the most frequently expressed (39). The anti-proliferative effect of SST analogs on the growth of the midgut carcinoids is unknown. A partial or complete responses were observed in <10% of the patients, while stabilization of tumor growth was noticed in 24–57% of the patients (6). Few data are available regarding the role of SST analogs in the treatment of gastrinomas. In a study of 15 malignant gastrinoma, in about 50% of these patients, octreotide had an anti-proliferative effect, including one patient with tumor regression and seven patients with tumor stabilization (mean period 25 months) patients (86). The long-acting SST analog octreotide-LAR was administered in a patient with multiple type A gastric carcinoids for a period of 9 months with a normalization of serum gastrin levels and permanent disappearance of the tumor (87). Fykse et al. treated five patients with hypergastrinemia and gastric carcinoids for a period of 1 year with monthly injections of octreotide-LAR with a significant reduction in tumor load, entero-chromaffin-like cells (ECL) cell density, and normalization of circulating chromogranin A levels, indicating a possible direct anti-proliferative effect of the treatment (88). These results suggest that the SST analogs could have an important anti-proliferative effect. However, data on the effect of SST analogs on tumor growth in patients with gastric carcinoids type C or poorly differentiated endocrine carcinomas are scanty. In poorly differentiated gastric carcinomas, treatment with SST analogs is not considered. As surgical excision is the definitive treatment of insulinoma, there are few contrasting data in the literature regarding the inhibitory effect of the SST analogs on the growth of these tumors. Grozinsky-Glasberg et al. have conducted a study regarding the effects of SST analogs on cell proliferation in the rat-derived insulinoma cell line (INS1). Their preliminary data show that octreotide has a significant inhibitory effect on cell proliferation, as assessed by cell counting and MTS assay, and on phosphorylation states of a number of proteins in the PI3K/Akt/mTOR pathway (89, 90). In his work, Vezzosi founded that despite achieving hypoglycemic control, insulinoma size remained unchanged or increased moderately despite normal blood glucose levels, concluding that SST analogs, as medical treatment is not sufficient to prevent tumor growth in patients with malignant insulinomas (40). In 2006, Romeo et al. reported a complete clinical remission with regression of the metastatic lesions in the liver after 1 year in a patient affected by metastatic insulinoma with severe hypoglycemia treated with octreotide-LAR (90). A more controversial area concerns the treatment of patients with non-functioning endocrine tumors of the pancreas as few studies have been published in these patients. The prospective German Sandostatin multicentre phase II trial investigated the effects of octreotide for 1 year on tumor growth in 103 patients and included 15 patients with diagnosed non-functional pancreatic tumors (79). Only 3 out of these 15 patients had a stable disease, in 8 patients a tumor progression occurred while the outcome of the remaining 4 patients was not clear. As previously reported, the SST analog efficacy depends on the tumor receptor expression patterns, but these are rarely assessed, even if there is evidence of better results on survival obtained with selective treatments. An anti-proliferative effect was achieved on hepatic metastatic cells in a patient with a carcinoid tumor, selected for the treatment with SST analogs after the immunohistochemical identification of the SSTRs 1, 2, and 5 subtypes expression on the neoplastic cell surface (91). A complete clinical remission with regression of the metastatic lesions in the liver after 1 year of treatment was observed in a patient affected by metastatic insulinoma with severe hypoglycemia treated with octreotide-LAR expressing at immunohistochemical analysis of tissue specimens a strong membrane immunoreactivity for SSTR 2 in both the primary nodule and the metastases (90). However, another study showed neither an anti-neoplastic effect nor an increase in survival percentage of treated patients (92). It has been reported that in glucagonoma patients there are no data available on their SSTR expression patterns (49). In 2006 we demonstrated, for the first time, a scattered immunopositivity for SSTRs in a case of malignant glucagonoma. We had access to polyclonal antibodies specifically targeted against SSTR5 and SSTR2 and we were therefore able to localize these two receptors in our histological sections. The immunopositivity was detected for both receptor subtypes in the membrane and in the cytoplasm of glucagonoma cells. We then treated our patient with a combination therapy consisting of the SST analog octreotide and interferon-a. The patient had a complete resolution of skin rash, normalization of plasma glucagon, chromogranin A, and neuron specific enolase levels and a metastatic disease stabilization. The patient’s quality of life significantly improved, and she was alive 40 months after debulking surgery (50).

**Table 4:** **Anti-proliferative effect of somatostatin analogues in patients with progressive disease**.

SSA	Dosagen	*n*	CR	PR	SD	PD	Reference
Lanreotide	3000 μg/day	22	0	1	7	14	(93)
Lanreotide	30 mg/2 weeks	35	0	1	20	14	(91)
Octreotide	600/1500 μg/day	52	0	0	19	33	(75)
Octreotide	1500/3000 μg/day	58	0	2	27	29	(28)
Lanreotide	15000 μg/day	24	1	1	11	11	(93)
Octreotide	600 μg/day	10	0	0	5	5	(74)
Octreotide median dose of 250 μg three times daily		34	0	1	17	0	(76)
Octreotide-LAR 30/lanreotide SR 60 mg/28 days		31	0	0	14	4	(77)
Total		256	1	6	115	105	
Percentage (%)			0.3	2	45	41	

*CR, complete remission; PR, partial remission; SD, stable disease; PD, progressive disease*.

## The Effects of Higher than Usual Dose of SST Analogs

It was suggested that higher than usual dose of SST analogs treatments (>3.000 μg/day) may promote the anti-proliferative effect, especially in those patients responding to standard doses (2, 17, 18, 83, 94, 95). A high-dose treatment with lanreotide (up to 12 mg/day) produced tumor size reduction in 5% and stabilization in 70% of the 19 patients. An induction of apoptosis in the tumors was observed in responding patients, a phenomenon not seen with regular doses of SST analogs, but often produced by chemotherapeutic agents (67). Subcutaneously injections of 5 mg lanreotide three times a day for a period of 1 year produced one complete and one partial remission (PR) in 30 patients with functional midgut NETs; stable disease in 11 patients (36%) and progression of the disease after 3–12 months of treatment in 11 patients (68). The treatment with high-dose SST analogs induced apoptosis in neuroendocrine tumors, while this was not found during treatment with low-dose SST, in a study where biopsy specimens were taken before and during SST analog treatment (66). In a highly select group of patients with progressive disease, 47% of the patients demonstrated at least stable disease when treated with a high dose of lanreotide (3–5 g/day) (82). High-dose formula of octreotide has been recently reported to stabilize hormone production and tumor growth in 75% of patients with advanced midgut carcinoid tumors and progressive disease with stabilization for 6–24 months (83). These effects may be attributable to SSTR 2, which is the most frequently expressed subtype and/or SSTR 5, 1, and 3 that are also expressed (96, 97). Data from a study with ultra-high-dose octreotide pamoate (Onco-LAR; Novartis) at 160 mg intramuscularly every 2 weeks for 2 months followed by the same dose once monthly, appear to show some promise. Tumor size stabilization was obtained in 12 patients, a biochemical responses in 9 patients and/or stability in 11. No significant tumor reduction was noted. At 6 months, the median plasma concentrations of octreotide were 25–100 times higher than those obtained by using octreotide-LAR at regular doses. A significant inhibition of angiogenesis was also showed through the down-regulation of proliferative factors such as VEGF and fibroblast growth factor (14). The highest response rates were reported using octreotide in doses >30 mg/day or lanreotide in doses >5 mg/day (and up to 15 mg/day) (64). Tomassetti et al. have reported that after 1-year therapy, the tumor completely disappeared in three patients suffering from gastric carcinoid, two of whom were treated with lanreotide 30 mg i.m. every 10 days (98). In a recent paper it was reported that in patients with Hashimoto’s thyroiditis presenting ECL hyperplasia, considered a pre-neoplastic mark, the treatment with SST analog for 12 months resulted into the disappearance of ECL lesions (93). SST analogs can have a role in the treatment of digestive neuroendocrine tumors with low grades of malignancy, a low cellular proliferation index and high specific receptorial density *in vivo* as reported by Bombardieri et al. (29). In a very complete and well design paper of Ferolla et al. patients with well-differentiated neuroendocrine tumors, were treated with long-acting octreotide (LAR), conventionally administered at a dose of 30 mg every 28 days; the end point of this study was to evaluate a different schedule of octreotide-LAR administration consistent with a shorter interval between administrations (21 days) in patients with a progressive disease at standard-dose interval. For this reason 28 patients who had tumor progression during therapy with LAR 30 mg every 28 days were enrolled. Clinical, biological, and objective tumor response was evaluated after LAR 30 mg every 21 days. Time to progression was also evaluated after LAR 30 mg every 21 days and compared to LAR 30 mg every 28 days. The treatment with LAR 30 mg every 21 days resulted in complete and partial control of clinical symptoms in 40 and 60% of cases, respectively. Circulating neuroendocrine markers were significantly decreased in 30% of cases. A stabilization of disease was obtained in 93% and objective response in 7%. The median time to progression was significantly longer by using the shortened interval of LAR administration as compared to the standard one (30 vs. 9 months, *p* < 0.0001) and the treatment was safe and well-tolerated. The authors conclude that the shortened schedule of LAR administration was able to re-institute control of clinical symptoms, to decrease level of circulating neuroendocrine markers and to increase time to progression in patients previously escaping from a standard schedule treatment (99).

## Somatostatin Analogs and Diagnostic/Therapeutic Nuclear Medicine

**Figure 2:**
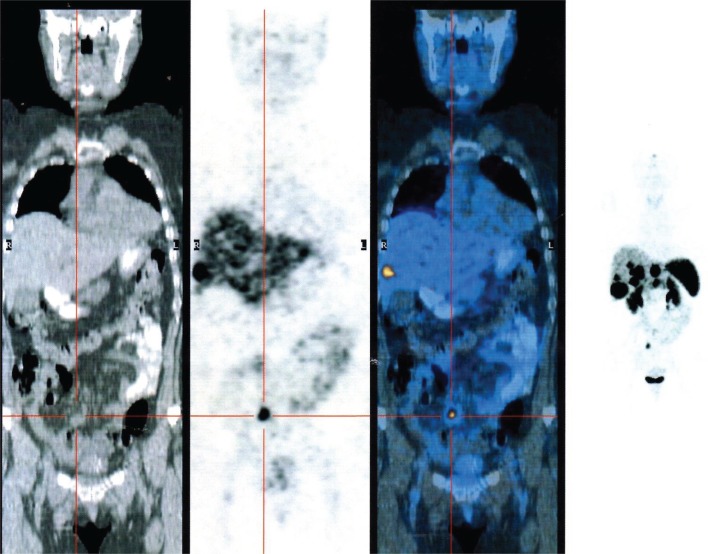
**PET gallium 68 DOTATOC showing the presence of multiple liver metastasis from neuroendocrine ileal tumor**.

Somatostatin receptors are able to form a receptor–ligand complex permitting the internalization and the accumulation of the radiopharmaceutical peptide inside the tumor using this procedures for diagnosis and radiometabolic treatments of these tumors (Figure 2). Peptide-receptor radionuclide therapy (PRRT) is an important treatment strategy for tumors that express adequate densities of SSTRs and has proven to be safe and effective. It was initially performed using indium-111 (24, 100). Recently, the development of SST peptides with higher receptor affinity conjugated with radio-metal labeling chelators, such as DOTA, which may be allow stable labeling with gallium, yttrium, or lutetium, changing the affinity profile for particular subtypes of SSTRs can permit new therapeutic options (101). Waldherr et al. evaluated the tumor response to targeted irradiation with the radiolabeled SST analog ^90^Y-DOTATOC in 41 patients with GEP-NET and bronchial tumors. They reported an ORR of 24%. For endocrine pancreatic tumors it was 36%. A complete remission was found in 2%, a PR in 22%, a minor response in 12%, stable disease in 49%, and progressive disease in 15% of patients. The treatment was well-tolerated and there was a significant reduction of symptoms and the 2-year survival time was 76 ± 16% (102). 177Lu DOTATATE [177Lu]DOTA-Tyr(3)-octreotate, a selective analog of SSTRs 2, in spite of its favorable affinity profile, at its maximum tolerated dose, it is limited by toxic effects on the kidney and bone marrow. Nevertheless, the results seem encouraging compared with historical therapeutic data (103). Kwekkeboom et al. obtained promising results using 177Lu DOTATATE [177Lu]DOTA-Tyr(3)-octreotate in 131 patients with NETs. A complete remission was observed in 2% of patients, a PR in 26%, a minor response in 19%, stable disease in 35%, and progressive disease in 18% of patients. Higher remission rates were positively correlated with high uptake on pre-therapy SSTRs imaging, whereas progressive disease was significantly more frequent in patients with extensive disease. Median time to progression was more than 36 months (24). The combination of ^90^Y- and ^177^Lu-labeled analogs (104) seems to have had superior antitumor effects when compared with either ^90^Y- or ^177^Lu-analog in animals presenting with tumors of various sizes. It has been reported that ^177^ Lutetium may be more effective for smaller tumors whereas ^90^Yttrium may be more effective for larger tumors (105, 106).

## Somatostatin Analogs and Interferon

The combination of SSAs and interferon (IFN) has been used in an effort to enhance the anti-proliferative effect of IFN therapy, to add the positive effect of SSAs on hypersecretory syndromes, and to reduce the dose of IFN and thus the number of IFN-related side-effects. Whether SST analogs and IFN show a synergistic effect on tumor growth and in carcinoid syndrome symptom management is matter of debate. The combination therapy with SST analogs and IFN is in fact limited by the small number of trials, with variable results. This combination seems of benefit in patients where the usual octreotide treatment failed to achieve a biochemical and symptomatic control (107).

## Conclusion

Neuroendocrine tumors of the gastroenteropancreatic (GEP-NETs) system comprise a rare group of malignant neoplasms. The SST analogs have been shown to be very useful for symptomatic and biochemical improvement in patients with these tumors while preclinical and clinical studies provide conflicting results on their antitumor effects. The mechanisms of these effects are unknown, but probably are in part due to direct effects on proliferative signaling pathways, activation of apoptosis, and effects on angiogenesis. Biological response to SST analogs depends on distribution and level of expression of SSTRs subtypes in tumors, and the expression of selective SSTR-signaling pathway molecules. The high density of SSTR 2 in endocrine tumors explains the use of SSTR 2 specific analogs in the diagnosis and treatment of these tumors. However, the role of SSTR 1, 3, and 5 appears to be of increasing interest. The development of new peptidic and non-peptidic SST analogs, subtype selective agonists, chimeric analogs, or pan-SST analogs will probably improve the diagnosis and treatment of GEP-NETs, which express SSTRs other than SSTR 2. The combination of SSAs and IFN seems of benefit in patients where the treatment with SST analogs alone failed to achieve a biochemical and symptomatic control while their synergistic effect on tumor growth is still unknown. The analysis of the SSTR status specifically for each patient, and studies of individual tumor biological behavior, might be of therapeutic interest and could help to optimize treatment especially in unresectable tumors. Peptide-receptor-targeted radiotherapy for advanced disease using radiolabeled octapeptide analogs appears to be a significant progress in the treatment of GEP-NETs but data are limited, mainly about the best time for its administration, or what is the most appropriate radioligand/combination to be used for each patient, and if and how the doses should be fractionated. Novel strategies based on SSTR 2 receptor gene transfer to target tumor growth and angiogenesis might offer new prospectives of therapeutic interest mainly to treat unresectable tumors. Prospective studies including large number of patients regarding the optimal dosage and modes of administration of SST analogs and the development of new slow release, SSTR subtype specific compounds are needed.

## Conflict of Interest Statement

The authors declare that the research was conducted in the absence of any commercial or financial relationships that could be construed as a potential conflict of interest.
